# Quantitative Analysis of Retinal Structure Using Spectral-Domain Optical Coherence Tomography in *RPGR*-Associated Retinopathy

**DOI:** 10.1016/j.ajo.2017.03.012

**Published:** 2017-06

**Authors:** James J.L. Tee, Joseph Carroll, Andrew R. Webster, Michel Michaelides

**Affiliations:** a UCL Institute of Ophthalmology, University College London, London, United Kingdom; b Moorfields Eye Hospital, London, United Kingdom; c Department of Ophthalmology and Visual Sciences, Medical College of Wisconsin, Milwaukee, Wisconsin

## Abstract

**Purpose:**

To quantify retinal structure and progression using spectral-domain optical coherence tomography (SDOCT) in patients with retinitis pigmentosa (RP) associated with retinitis pigmentosa GTPase regulator gene (*RPGR*) mutations.

**Design:**

Retrospective observational case series.

**Methods:**

Setting: Moorfields Eye Hospital, London, United Kingdom. Subjects: Both eyes of 32 patients. SDOCT follow-up period of >1 year (3.1 ± 1.4 years). Main Outcome Measures: Ellipsoid zone (EZ) width (EZW) and outer nuclear layer (ONL) and inner retinal layer (IRL) thickness measurements. Progression rates, interocular symmetry, and association with age and genotype were investigated.

**Results:**

Significant differences were observed between baseline and final measurements of EZW and ONL thickness, but not for IRL thickness. Baseline and final EZWs were 2438 ± 1646 μm and 1901 ± 1423 μm for right eyes (*P* < .0001); 2420 ± 1758 μm and 1922 ± 1482 μm for left eyes (*P* < .0001). EZW constriction rates were 176.6 ± 130.1 μm/year and 173.1 ± 146.8 μm/year for right and left eyes. ONL thinning rates were 2.58 ± 2.85 μm/year and 2.52 ± 3.54 μm/year for right and left eyes. Interocular differences in EZW and ONL progression were not significant (*P* = .8609 and *P* = .6735, respectively). Strong correlations were found between EZW constriction rates of right and left eyes (r_s_ = 0.627, *P* = .0002) and between EZW constriction and baseline EZW (r_s_ = 0.714, *P* < .0001). There was moderate negative correlation between EZW constriction and age (r_s_ = −0.532, *P* < .0001). Correlation between ONL thinning and age was not significant, as were differences between EZW and ONL progression rates with respect to genotype.

**Conclusions:**

This study provides SDOCT progression rates for *RPGR*-associated RP. There is overall interocular symmetry with implications for future treatment trials where 1 eye could serve as a control.

Retinitis pigmentosa (RP) is a group of genetically diverse disorders characterized initially by nyctalopia, progressive visual field constriction, and finally decreased central vision in the advanced stage. The prevalence of RP is estimated to be 1:3000, with 30%–40% of cases inherited via an autosomal dominant retinitis pigmentosa, 45%–60% via an autosomal recessive route (ADRP), and 5%–15% as an X-linked (XL) trait.^1^, ^2^, ^3^, ^4^, ^5^ *RPGR* mutations account for 70%–80% of XLRP^6^, ^7^, ^8^ with *RP2* variants accounting for a further 6%–20%.^7^, ^8^, ^9^, ^10^, ^11^ *RPGR*-associated RP is particularly severe, with an early onset of disease in childhood. There is marked phenotypic heterogeneity in the condition, and rate of disease progression varies between affected individuals.^12^ *RPGR* mutations have also been associated with other clinical phenotypes, including cone-rod dystrophy, macular atrophy, and, rarely, syndromic XLRP (OMIM*312610).^12^, ^13^ Less phenotypic variation is seen with *RP2*, with XLRP being the only associated clinical phenotype (OMIM*300757).^14^ Unlike *RPGR*-associated RP, early macular involvement with reduced visual acuities has been described as a distinguishing feature in *RP2-*associated RP.^8^, ^15^ An earlier study, however, did not find differences between *RP2* and *RPGR* phenotypes.^16^ Despite overlapping symptoms of *RPGR*- and *RP2*-associated RP, the renewed effort in recent years to distinguish between genetic mutations that give rise to disease phenotypes is crucial with the advent of gene-directed therapy and other novel interventions.^17^

Spectral-domain optical coherence tomography (SDOCT) is an established modality for studying retinal structure in vivo. In subjects with RP, there exists a continuum from apparently healthy central retinal tissue to diseased peripheral retina (termed the “transition zone”), which can be categorized into distinct regions based on retinal morphology. Jacobson and associates described 4 subzones, with emphasis on the appearance of the photoreceptor outer nuclear layer (ONL) and inner retinal layers, from (1) normal retina centrally to (2) regions of ONL thinning, (3) ONL loss accompanied by inner nuclear layer thickening, and finally (4) retinal atrophy at the periphery.^18^ Hood and associates included an assessment of the photoreceptor outer segment (OS) in addition to ONL thickness in their definition of the transition zone.^19^ The structurally intact foveal center extends centrifugally into a region characterized by OS thinning, then into a region where there is both OS and ONL layer thinning, followed by total loss of OS and ellipsoid zone (EZ) band, and finally into a region where the ONL layer has completely thinned down to lie at an asymptote to the retinal pigment epithelium (RPE).^19^

Assessment of the rate of progression in XLRP has been undertaken by measurement of EZ constriction over time on line scans,^20^, ^21^, ^22^ and to a lesser extent by calculating global average retinal thickness derived from volume scan segmentation of photoreceptor and ONL layers.^21^ The reliability of these metrics to quantify disease progression has also been explored.^21^ However, these studies do not distinguish between *RPGR* and other genetic causes of XLRP, and are potentially limited by eye selection bias.^20^, ^21^, ^22^

The use of EZ width metrics as a marker for disease severity and progression in XLRP correlates well with retinal function, with intact foveal EZ associated with good visual acuity^23^ and the length of intact EZ closely correlated to the extent of the preserved visual field.^24^ A drop of 8 dB in retinal sensitivity between regions where EZ is present compared with adjacent regions of EZ loss has been described^25^ and substantiated by the discovery that the greatest rate of retinal sensitivity decline occurred in regions on either side of EZ band disappearance, as opposed to the central 10 degrees, where the photoreceptors are still relatively healthy.^26^ ONL thinning is also noted to occur in areas of declining sensitivity.^18^, ^26^, ^27^ Regions of ONL loss are often accompanied by inner retinal thickening, as reported in ADRP associated with *RHO*, in Usher syndrome type 1 associated with *MYO7A*, and in XLRP associated with *RPGR*.^18^, ^27^, ^28^ However, others have observed that any increase in inner retinal thickness is owing to thickening of the nerve fiber layer (NFL).^19^, ^29^ There have also been observations that NFL thinning or a combination of thickening and thinning can occur.^30^, ^31^

*RPGR* is an important focus for gene therapy research, with recent successes demonstrated in animal models^32^, ^33^ and human clinical trials in preparation.^33^ It is anticipated that SDOCT imaging will be a crucial modality in the assessment of retinal structure pre- and post intervention. The current lack of natural history data in individuals harboring pathogenic mutations in *RPGR*, however, poses a limiting factor. As such, the aims of this study were to investigate and quantify the following in patients with molecularly confirmed *RPGR* RP: (1) EZ width (EZW) and ONL and inner retinal layer (IRL) thickness on SDOCT; (2) progression rates of the aforementioned metrics; (3) interocular symmetry of baseline measurements and progression rates; (4) association between baseline measurements and age; (5) association between progression rates and age; and (6) association between progression rates and baseline EZW. This study specifically describes findings in patients with *RPGR*-associated RP.

## Methods

This retrospective observational SDOCT study was registered and approved by the R&D Department of Moorfields Eye Hospital, London, United Kingdom, with adherence to the Declaration of Helsinki kept throughout the study. A search of the Moorfields Inherited Eye Disease Database was last performed on April 7, 2016, to identify male subjects with likely disease-causing sequence variants in *RPGR* resulting in an RP phenotype, who have previously attended the Medical Retina Genetics clinics at Moorfields Eye Hospital, London, United Kingdom. Patients with SDOCT follow-up of more than 1 year and in whom the EZ was located within the imaging borders were selected for inclusion.

Bidirectional sequencing to test for mutations in *RPGR* exons 1–14 and Open Reading Frame 15 (ORF15) were performed for all patients at the Central Manchester University Hospitals Genomic Diagnostics Laboratory, UK. Mutations that were confirmed likely to be pathogenic either have been reported in the literature or, in the case of novel mutations, were predicted to result in the disruption of normal protein translation. We additionally cross-referenced the mutations with the *RPGR* variant database that is maintained by the Medical Research Council Human Genetics Unit, Edinburgh, UK,^34^, ^35^ to corroborate novel mutations. Mutations were also entered into Mutalyzer 2.0.23 (https://mutalyzer.nl/) to confirm disruption of protein translation.^36^

**Figure 1:**
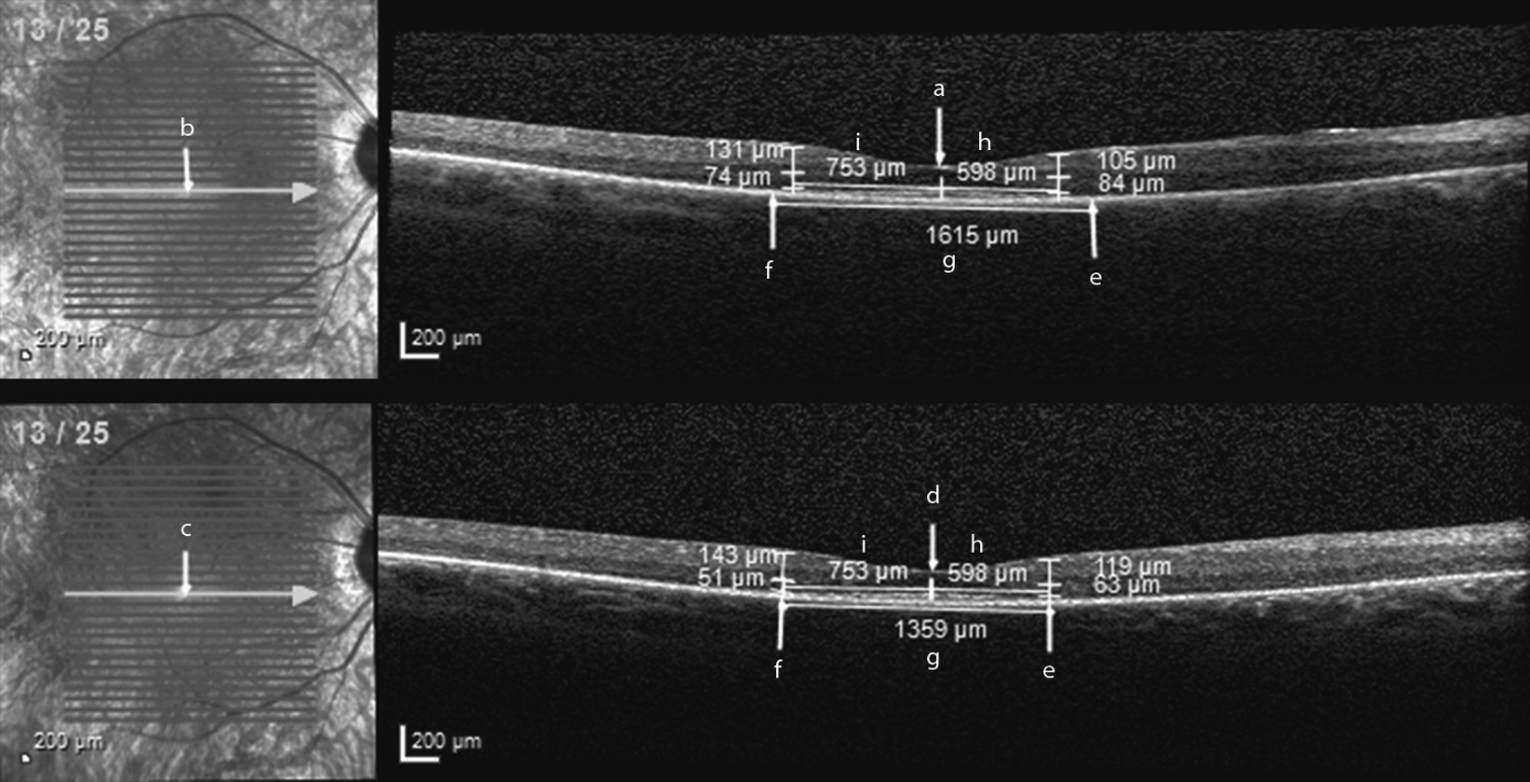
Baseline (Top) and final (Bottom) optical coherence tomography (OCT) images of a patient with *RPGR*-associated retinopathy, taken with a 1.5-year interval. Ellipsoid zone width was 1615 μm at baseline and 1359 μm on final OCT. Nasal outer nuclear layer (ONL) thickness was 84 μm at baseline and 63 μm on final OCT. Nasal inner retinal layer (IRL) thickness was 105 μm at baseline and 119 μm on final OCT. Temporal ONL thickness was 74 μm at baseline and 51 μm on final OCT. Temporal IRL thickness was 131 μm at baseline and 143 μm on final OCT.

Ophthalmic photographers acquired images on the Spectralis OCT device (Heidelberg Engineering, Heidelberg, Germany). Where available, horizontal high-resolution line scans with automatic real-time tracking were selected for analysis and supplemented with transfoveal line scans obtained from horizontal volume scans. Images were analyzed with vendor software (Heidelberg Eye Explorer Region Finder Version 2.4.3.0) in a 1:1 micron setting with the following method: The foveal center is marked on the baseline transfoveal OCT image (Figure 1, annotation ‘a’) and the corresponding point marked on the accompanying baseline HRA fundus image (Figure 1, annotation ‘b’). The baseline HRA overlay is copied and pasted on the final HRA fundus image as per vendor software (Figure 1, annotation ‘c’). The vertical OCT marker position on the final image is then adjusted to correspond to that shown on the final HRA image (Figure 1, annotation ‘d’). This ensures the same location is selected as the foveal center on both baseline and final OCT images, and is particularly important when locating corresponding positions for follow-up measurements of retinal thickness. Next, nasal and temporal EZ edges on baseline and final OCTs are marked with the arrow tool (Figure 1, annotations ‘e’ and ‘f’). This is taken at the point of EZ disappearance into the proximal RPE border. EZW is measured by drawing a straight line tangential to the distal RPE border with the caliper tool for both images (Figure 1, annotation ‘g’). Horizontal distances between foveal center and both nasal and temporal EZ edges are measured on the final OCT (Figure 1, annotations ‘h’ and ‘i’) and the same distances are subsequently marked out on the baseline OCT (Figure 1, annotation ‘h’ and ‘i’). This step enables retinal thickness measurements to be taken at corresponding retinal locations on both baseline and final OCT images (ie, at the “transition points”). Vertical thickness measures of both the ONL and IRL are taken orthogonal to the retina at the nasal and temporal transition points of both baseline and final OCT images.

ONL thickness was measured from the border along the outer plexiform layer/inner nuclear layer (OPL/INL) to the external limiting membrane (ELM). The OPL/INL border was used for ONL thickness measure instead of the outer nuclear layer/outer plexiform layer (ONL/OPL) border, as the OPL/INL border is more clearly demarcated than the ONL/OPL border, which also contains Henle fibers. IRL thickness was measured from the ganglion cell layer/nerve fiber layer (GCL/NFL) border to the OPL/INL border. As such, the IRL thickness measure contains the GCL, IPL, and INL but not the NFL. Image magnification is optimally adjusted throughout to facilitate the identification of retinal borders and layers.

Statistical analysis was performed with XLSTAT version 2014.6.02 (Addinsoft, New York, New York, USA) software. Data are expressed as mean values, with standard deviation and 95% confidence intervals provided where stated. Differences in baseline and final EZW, ONL, and IRL measurements were assessed for significance with the Wilcoxon signed rank test. Intraocular and interocular differences in EZW and ONL progression rates were also investigated with the aforementioned test. Spearman correlation coefficient was calculated to investigate interocular associations of symmetry for baseline EZW and ONL thickness; interocular symmetry of EZW and ONL progression rates; association between age and baseline EZW and ONL thickness; association between baseline EZW and progression rate; and association between age and EZW progression rate. Study eyes were categorized into 2 groups, those with mutations in exons 1–14 or ORF15, prior to conducting a Mann-Whitney test for the purpose of investigating potential differences in progression rates with respect to genotype. Study eyes were further recategorized into 2 groups based on predicted effects of their mutations: those with null allele mutations (premature stop codons or frameshifts leading to premature stop codons in exons 1–14) or those with mutations likely to result in translation of a variant protein product (missense mutations and mutations in ORF15).^37^ Eyes of 2 patients with splice site mutations were excluded from this analysis owing to the greater challenge in predicting their effects.^37^ Significance level alpha was set at 0.05 and 2-tailed *P* values were calculated for all statistical testing.

## Results

**Figure 2:**
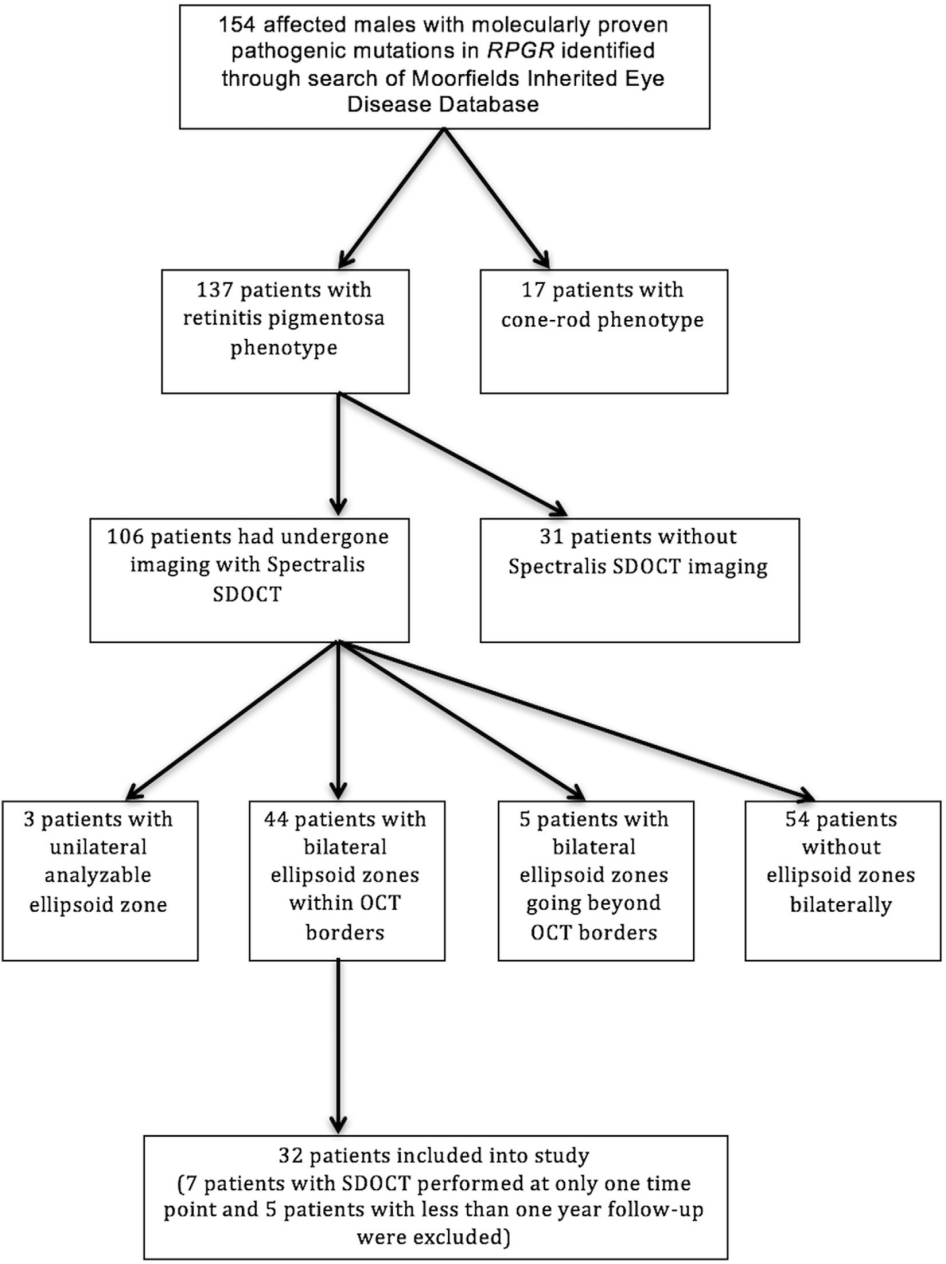
Flowchart documenting the recruitment of subjects with *RPGR*-associated retinopathy as per the inclusion and exclusion criteria.

Thirty-two patients had both eyes that met the inclusion criteria, as shown on the flowchart in Figure 2. Age at presentation, as calculated from time of birth to time of baseline image acquisition, was 19.1 ± 7.9 years. Out of the 32 patients, 22 were white, 2 South Asian, 1 black, and 1 Middle-Eastern, and 6 others had unspecified ethnic backgrounds. Fourteen patients had pathogenic mutations in *RPGR* exons 1–14, and 18 patients had mutations within *RPGR* ORF15. As shown in Table 1, 7 out of 10 novel mutations lay within *RPGR* exons 1–14. All novel mutations were predicted to result in premature termination. The interval between baseline and final OCT scans was 3.1 ± 1.4 years. Baseline and final EZW, ONL, and IRL thickness values with corresponding *P* values calculated from intraocular comparisons are provided in Table 2.

**Table 1:** *RPGR* Mutations of All Study Patients and Their Predicted Effects on Protein Translation

Exon	Genetic Mutation (Based on Accession Number NM_001034853.1)	Effect
Exons 1–14	Exon 6–11 deletion*	Premature termination
	Exon 7 deletion*	Premature termination
	c.799G>A	Missense mutation
	c.836_934+1276del	Exon/intron 8 splice site
	c.880delG*	Premature termination
	c.891_892delAA	Premature termination
	c.914dupA*	Premature termination
	c.1234C>T	Premature termination
	c.1243_1244delAG*	Premature termination
	c.1387C>T*	Premature termination
	c.1429G>T*	Premature termination
	c.1572+1G>A	Exon/intron 13 splice site
ORF 15	c.2045_2046dupGT*	Premature termination
	c.2238delA*	Premature termination
	c.2245G>T	Premature termination
	c.2384delA	Premature termination
	c.2405_2406delAG	Premature termination
	c.2476_2477delAG	Premature termination
	c.2586_2587delGG	Premature termination
	c.2601_2602delGG	Premature termination
	c.2625dupA	Premature termination
	c.2907_2910delAGGA	Premature termination
	c.2993_2997delAAGGG	Premature termination

ORF = open reading frame.

Novel mutations are indicated by an asterisk.

**Table 2:** Values for Baseline and Final Optical Coherence Tomography–Derived Metrics for Study Subjects With *RPGR*-Associated Retinopathy

	Right Eyes (N = 32)			Left Eyes (N = 32)		
	Baseline (Mean ± SD)	Final (Mean ± SD)	Intraocular Comparison	Baseline (Mean ± SD)	Final (Mean ± SD)	Intraocular Comparison
EZW (μm)	2438 ± 1646	1901 ± 1423	*P* < .0001	2420 ± 1758	1922 ± 1482	*P* < .0001
Nasal ONL thickness (μm)	66.4 ± 11.9	59.1 ± 10.4	*P* < .0001	66.6 ± 11.3	61.0 ± 10.9	*P* = .0036
Temporal ONL thickness (μm)	66.3 ± 9.0	59.4 ± 8.8	*P* = .0006	64.4 ± 11.1	58.0 ± 8.6	*P* = .0003
Nasal IRL thickness (μm)	105.8 ± 46.5	104.0 ± 49.5	*P* = .9851	110.8 ± 45.2	103.2 ± 45.0	*P* = .0042
Temporal IRL thickness (μm)	102.4 ± 45.9	105.1 ± 44.8	*P* = .0958	102.7 ± 43.6	99.1 ± 42.9	*P* = .1238

EZW = ellipsoid zone width; IRL = inner retinal layer; OCT = optical coherence tomography; ONL = outer nuclear layer; SD = standard deviation.

Two-tailed Wilcoxon signed rank test was used to investigate differences between baseline and final values with significance level alpha set at 0.05.

**Figure 3:**
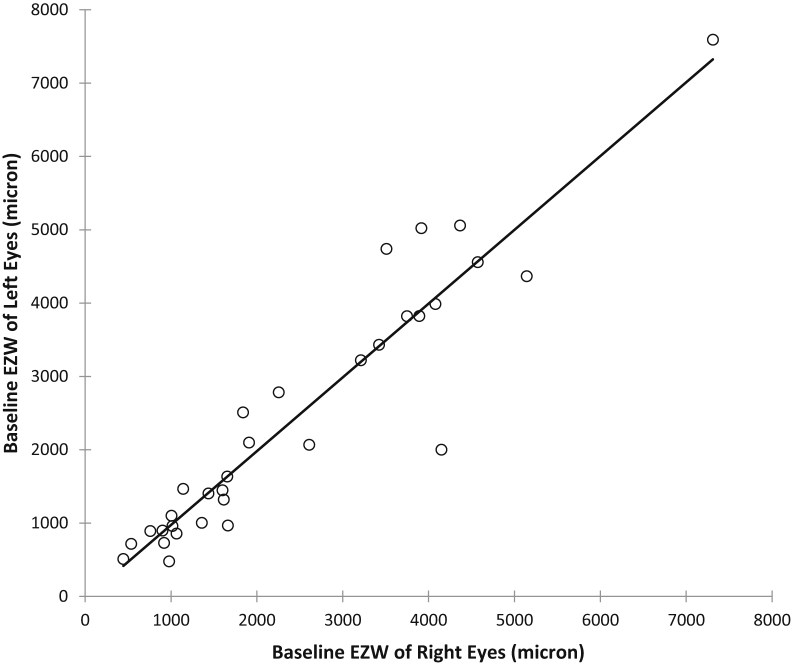
Scatterplot of interocular ellipsoid zone width (EZW) at baseline for all study subjects with *RPGR*-associated retinopathy, right eyes corresponding to left eyes. Spearman correlation coefficient, r_s_ = 0.931, *P* < .0001 indicates a very strong and significant interocular correlation.

As shown in Figure 3, a very strong and significant correlation is present between baseline EZW of right and left eyes (Spearman correlation coefficient, r_s_ = 0.931, *P* < .0001). Baseline ONL thickness correlated moderately between right and left eyes (r_s_ = 0.509, *P* = .0033).

A significant negative correlation was found between baseline EZW and age, indicating a smaller baseline EZW in older eyes (r_s_ = −0.594, *P* < .0001). Correlation between baseline ONL thickness and age was weak (r_s_ = 0.250, *P* = .0467).

As shown in Table 2, the differences between baseline and final values of EZW and ONL thickness measurements were significant, in contrast to the baseline and final IRL values. EZW and ONL progression rates together with corresponding *P* values obtained from intraocular and interocular comparisons are provided in Table 3. Progression rates for IRL were not calculated, as baseline and final values for this metric did not differ significantly. EZW constriction occurred at a rate of 176.6 ± 130.1 μm/year for right eyes and 173.1 ± 146.8 μm/year for left eyes. Average rate of ONL thinning was 2.58 ± 2.85 μm/year and 2.52 ± 3.54 μm/year for right and left eyes, respectively. Intraocular and interocular differences in EZW and ONL progression rates were not statistically significant.

**Table 3:** Ellipsoid Zone Width and Outer Nuclear Layer Progression Rates for Study Subjects With *RPGR*-Associated Retinopathy

	Right Eyes (N = 32)		Left Eyes (N = 32)		Interocular Comparison
	Mean (± SD)	95% CI for Mean	Mean (± SD)	95% CI for Mean	
Rate of EZW constriction (μm/yr)	176.6 ± 130.1	129.7–223.5	173.1 ± 146.8	120.1–226.0	*P* = .8609
Rate of nasal ONL thinning (μm/yr)	2.71 ± 3.83	1.33–4.10	2.57 ± 5.21	0.70–4.45	*P* = .1619
Rate of temporal ONL thinning (μm/yr)	2.44 ± 4.01	0.99–3.88	2.48 ± 4.70	0.78–4.17	*P* = .8465
Average rate (nasal and temporal) of ONL thinning (μm/yr)	2.58 ± 2.85	1.55–3.60	2.52 ± 3.54	1.25–3.80	*P* = .6735
Intraocular comparison between nasal and temporal ONL thinning rates	*P* = .9922		*P* = .4716		

EZW = ellipsoid zone width; ONL = outer nuclear layer.

Positive values signify EZW constriction or ONL thinning. Two-tailed Wilcoxon signed rank test was used for statistical testing with significance level alpha set at 0.05.

**Figure 4:**
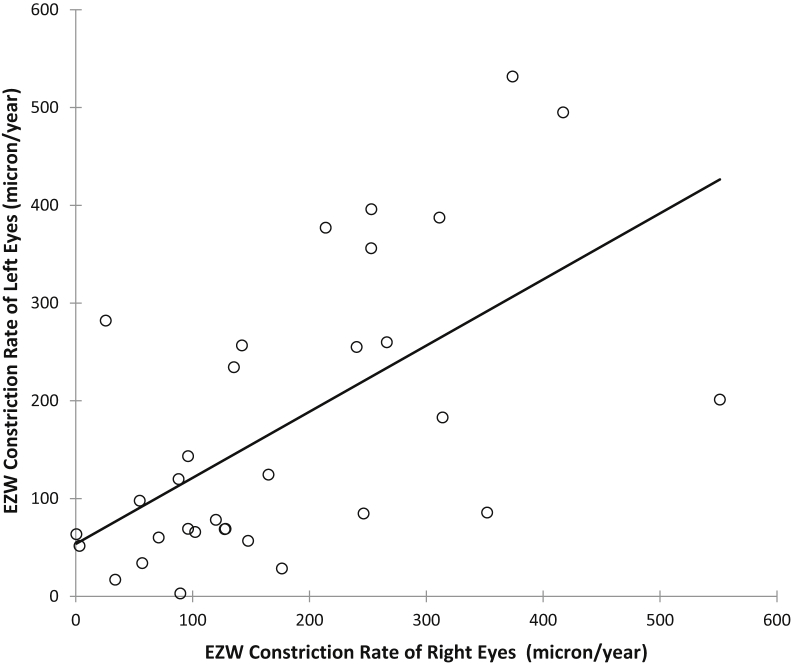
Scatterplot of interocular ellipsoid zone width (EZW) constriction rates for all study subjects with *RPGR*-associated retinopathy, right eyes corresponding to left eyes. Spearman correlation coefficient, r_s_ = 0.627, *P* = .0002 indicates a strong and significant interocular correlation in EZW progression.

A scatterplot of interocular EZW progression rates is shown in Figure 4. A significant and strong correlation is found in the EZW progression rates between right and left eyes (r_s_ = 0.627, *P* = .0002), but not for ONL thinning rates between right and left eyes (r_s_ = 0.172, *P* = .3432).

**Figure 5:**
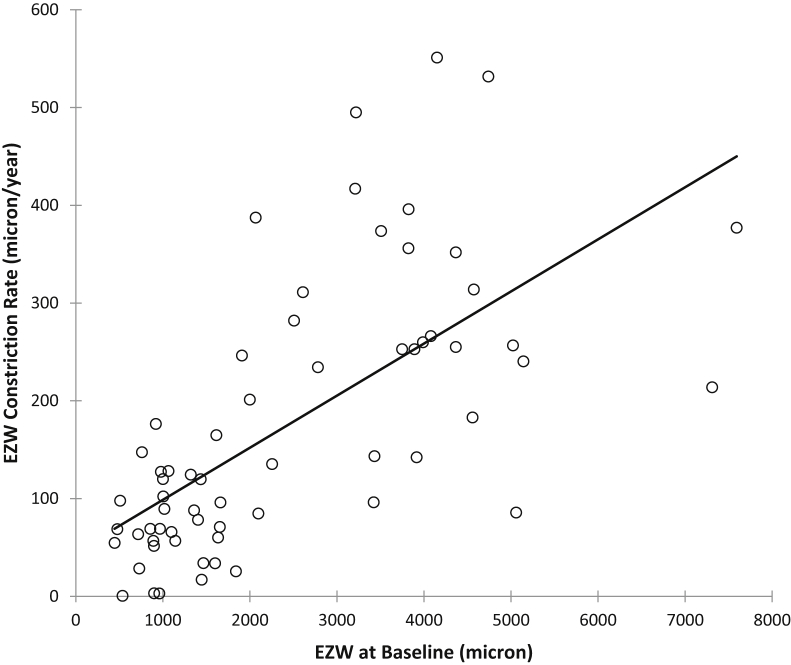
Scatterplot of ellipsoid zone width (EZW) constriction rates vs EZW at baseline for study subjects with *RPGR*-associated retinopathy. There is a strong and significant correlation indicating greater progression rates in eyes with larger EZW (r_s_ = 0.714, *P* < .0001).

The association between rates of disease progression with respect to baseline EZW and age were investigated. Figure 5 shows a scatterplot of EZW constriction rates vs. their baseline EZW. There is a strong and significant correlation, indicating that progression as measured by rate of EZW constriction is greater in eyes with larger baseline EZW (r_s_ = 0.714, *P* < .0001). There was a moderate and negative correlation between EZW constriction rates with age (r_s_ = −0.532, *P* < .0001). Correlation between ONL thinning rates with age were, however, weak and not statistically significant (r_s_ = −0.172, *P* = .1748).

To investigate potential differences in EZW and ONL progression rates as conferred by genotype, eyes were categorized into 2 groups: those with either exon 1–14 mutations or mutations in ORF15. Statistical testing with the Mann-Whitney test indicated that differences were not significant (EZW constriction rate for the exon 1–14 group was 172.47 ± 149.38 μm/year compared to 176.63 ± 129.87 μm/year for the ORF15 group, *P* = .7117. ONL thinning rate for the exon 1–14 group was 1.73 ± 2.45 μm/year compared to 3.19 ± 3.56 μm/year for the ORF15 group, *P* = .0667).

Eyes were recategorized into 2 groups to aid investigation of potential differences in EZW and ONL progression rates as conferred by mutation type: those with null alleles or those with mutations that give rise to variant protein products. Statistical testing with the Mann-Whitney test indicated that differences for EZW constriction rate were insignificant between the groups (EZW constriction rate for the null allele group was 199.00 ± 158.12 μm/year compared to 171.35 ± 128.43 μm/year for the variant protein product group, *P* = .5861). Differences in ONL thinning rate were borderline significant, with a lower ONL thinning rate for the null allele group of 1.69 ± 2.44 μm/year compared to 3.20 ± 3.47 μm/year for the variant protein product group, *P* = .0437.

## Discussion

We present the first comprehensive series describing progression rates in both eyes of molecularly proven *RPGR*-associated RP patients using metrics derived from the transition zone as characterized by Hood and associates.^19^ EZW annual constriction rates of 176.6 μm/year (7.98%/year) for right eyes and 173.1 μm/year (7.66%/year) for left eyes in our study are comparable to that previously reported for XLRP.^20^, ^22^ Birch and associates found a decrease of 248 μm/year (7%/year) in their cohort of 28 XLRP patients with a cohort baseline EZW of 3576 μm. Details regarding causative genes for disease were not provided, nor was justification on how they chose which eye of their subjects for analysis.^20^

Our EZW annual constriction rate is less than that described by Cai and associates of 289 μm/year (9.6%/year) in their cohort of 26 XLRP patients, 25 of whom had mutations in *RPGR*, with no information provided on the exact sequence variants.^22^ Uniocular data were analyzed in their study, with ocular selection made on the basis of the eye with a clearer EZ band for analysis, with a mean baseline EZW of 3410 μm. Thirty-three patients with ADRP were also studied for comparison and a slower annual progression rate of 115.6 μm/year was found (3.4%/year).^22^

The baseline EZW of 2438 μm and 2420 μm for right and left eyes, respectively, indicates that our cohort as a whole is composed of patients with relatively more advanced disease, in comparison with the other 2 aforementioned studies. The negative correlation of baseline EZW with age in our cohort is consistent with increasing disease severity with age as a consequence of retinal degeneration. The possibility of decreasing rates of progression with advancing disease as the transition zone approaches the fovea has previously been raised.^20^, ^38^ This hypothesis is supported by our finding of a strong and significant correlation between rate of EZW constriction and baseline EZW, and is reflected in a further analysis of EZW constriction rates subsequent to the division of our cohort into 2 age groups, each falling on either side of the mean presentation age. Subjects under 19 years of age have a greater rate of EZW constriction compared with those 19 years or older (right eyes: 198.5 μm/year for <19 years, 154.7 μm/year for ≥19 years; left eyes: 228.3 μm/year for <19 years, 117.78 μm/year for ≥19 years). Knowledge of disease duration may serve as a better correlate of disease severity and progression rates. It will likely, however, prove challenging to precisely calculate disease duration, as one would need to establish the exact time when symptoms of nyctalopia first occurred/retinal dysfunction-degeneration commenced, in the context of the early onset of disease in childhood typical of *RPGR*-associated RP. In our study, we have used numerical age as an approximation for disease duration.

To the best of our knowledge, this is the first study to characterize disease progression in both eyes of subjects with molecularly proven *RPGR-associated* RP with a follow-up duration of 3.1 years. The strong and significant correlation in baseline interocular EZW and interocular EZW rates of progression, together with a lack of significant differences in rates of progression, imply symmetry between eyes.

The direct measurement of EZW on transfoveal line scans is believed to be the most “practical” and “effective” method of assessing progression over other methods of quantifying thickness of various retinal layers across an entire volume scan.^21^ This latter method involves the segmentation of retinal layers, for example the outer nuclear layer for all B-scans that form the volume scan to derive a “global” average thickness value that encompasses the entire transition zone from healthy to diseased retina. It has, however, been demonstrated that any measurable change with this method is minimal at best.^21^ This is not unexpected, as region C of the transition zone where EZ disappearance occurs^19^ is where disease is most active, as exemplified by the sharp drop in retinal sensitivity compared to the central and peripheral regions, which would be included in a global average thickness value, likely resulting in the dilution and loss of the potential identification of progression.

Herein we have described an alternative attempt to quantify ONL thickness by taking serial ONL thickness measures at specific points, which we term “transition points,” that are located within region C of the transition zone where photoreceptor degeneration is actively occurring. The technique of foveal registration allows measurements to be made at similar locations on sequential OCT images. To the best of our knowledge, this method has not been undertaken in previous studies. The ability to optimally magnify images to enable clearer delineation of retinal layers without pixellation was preserved, as images were not exported out of the vendor software. Despite this, we could not detect any significant correlation between ONL thinning rates and age, or between right and left eyes. This may be owing to the inclusion of OPL and Henle fibers (which is dominated by fibers from the foveal cones at the parafovea) in our ONL measurements. Likewise, we cannot rule out the effect this had on the findings of borderline significance in the lower ONL thinning rate for the null allele group, despite insignificant differences in EZW constriction rates. This may, however, warrant further investigation in future studies.

In conclusion, we have provided structural data on progression rates for *RPGR*-associated RP as obtained from OCT-derived metrics. The finding that overall rate of progression in both eyes is similar has implications for future gene therapy trials where 1 eye could potentially serve as a control.

## Figures and Tables

**Figure:**
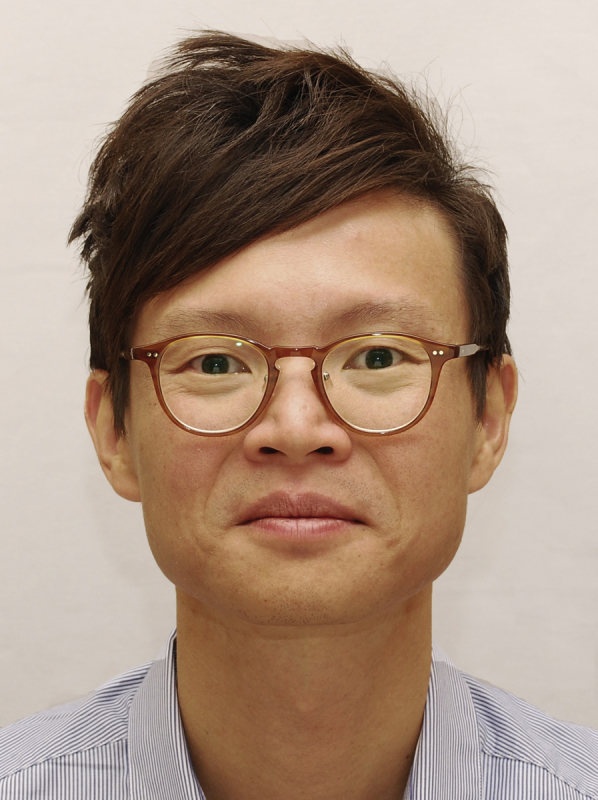
Dr James J.L. Tee, MBBS, FRCOphth is a clinical research fellow with the University College London Institute of Ophthalmology and Moorfields Eye Hospital, London, United Kingdom. Dr James J.L. Tee graduated with a medical degree from Imperial College, London, United Kingdom in 2005 and completed his ophthalmology residency at Moorfields. He was admitted in 2013 as a fellow of the Royal College of Ophthalmologists, United Kingdom. His current research interests involve *RPGR*-associated retinitis pigmentosa and retinal imaging.
